# The Book World of Medicine and Science

**Published:** 1897-04-03

**Authors:** 


### THE BOOK WORLD OF MEDICINE AND SCIENCE.

Functional Nervous Disorders in Women. By T. J. McGillicuddy, A.M., M.D. (London: Baillière, Tindall, and Cox, 1896.)

The ground covered by this treatise includes that branch of gynæcology which is usually but cursorily dealt with in text-books and manuals on the subject of diseases of women. Gynæcological conditions, which are unaccompanied by demonstrable pathological lesions, have heretofore found few serious exponents; and, for this reason, the profession will welcome the results and observations of a physician who has devoted himself to research in what may be called its medical side. Reflex and central neuroses, their diagnosis, pathology, and treatment constitute the bulk of the book; but, although by no means exhaustive or complete, the more familiar conditions are severally dealt with, and, to a very praiseworthy extent, a systematic and orderly arrangement have been adopted. Thus, to a certain degree, it may serve as a book of reference for the practitioner when in difficulty over special cases. But where its real value will probably be found to lie will be that it is a most readable dissertation on the neuroses of women. It is decidedly an "arm-chair" book, full of interesting facts, striking illustrations, and useful hints, and the reader will not fail to derive advantage from the point of view of the general treatment and diagnosis of the more frequent gynæcological ailments met with in general practice, and which do not call for surgical interference. Perhaps, the least satisfactory portion is that which deals with the general treatment, and to this section nearly a third of the entire space has been devoted. In failing in this respect Dr. McGillicuddy finds himself in excellent company, and it is an experience which is met with every day, when any particular line of treatment is put to the practical test in gynæcological practice. In dealing with areas of superficial pain, which correspond to disturbances in internal structures, no mention is made of the work of Dr. Head, whose researches in this department have attracted a good deal of attention in this country. The scientific accuracy of the whole would be held in greater respect if such expressions as "stomach cough" and "biliousness" were in several instances deleted: although popular and familiar terms among lay people, to most medical men they are terms only to use at the bedside. With these minor exceptions the book must be considered as one of great value.

Death and Sudden Death. By P. Brouardel, Professor of Medical Jurisprudence, and Dean of the Faculty of Medicine, Paris. Translated by F. Lucas Benham, M.D., B.S., M.R.C.P. (London: 1897. Baillière, Tindall, and Cox. Pp. 270. Price 10s. 6d.)

To characterise a book on Death as fascinating may appear to argue a gruesome predilection on the part of the reviewer; nevertheless, it must be admitted that Professor Brouardel's treatment is such as to make one forget the grimness of his subject. The terse picturesqueness of diction which has been a tradition in French scientific writing since the days of Descartes is here seen at its best, and loses nothing of its charm in Dr. Benham's elegant version. When it is further remembered that, as director of the Morgue, Dr. Brouardel has had a medico-legal experience which has been unique both in extent and variety, it will be seen that the circumstances attending the production of this book are most happily combined. The work comprises two courses of lectures delivered in the Paris Faculty of Medicine, the first treating of the signs of death, the second of the causes of sudden death, a subject which has been unduly neglected by English writers. Under the first heading the author has very little that is new to tell us; the especial value of this part is the scientific and judicious manner in which he presents the problems, and indicates the means at our disposal for solving them. We cannot, however, agree with his view that rigor mortis is only an early stage of putrefaction, for a dead muscle will become rigid if micro-organisms are rigorously excluded, and even in a vacuum; nor can we accede to his statement (page 102) that a smell is infectious. The most interesting chapter in this series is the one in which an account is given of Mégnin's valuable researches into post-mortem entomology. This careful observer divides the work of the "labourres of death" into four stages, and is able by an examination of the insects infesting the corpse to establish many important facts relating to the time of death and the subsequent conditions of environment. The second and longer section of Dr. Brouardel's book is devoted to an exhaustive account of the causes of sudden death, classified under the headings of the different organs which have given way, and illustrated by details of many interesting cases. Some such account was much needed, and we cannot conceive of a more admirable exposition of the subject than that under notice. The author lays, perhaps, a little too much stress on "inhibition" as a cause of death, but he deserves much praise for the outspoken expression of his disbelief in that much-quoted malady, simple primary pulmonary congestion. He holds that pleurisy is the most common pulmonary cause of sudden death. He shows also, in a very interesting manner, how death from acute alcoholism is really the result of cold. The book is handsomely produced, and the translation, as we have already said, on the whole excellent. There is, however, an amusing slip on page 27, where the respiratory capacity of M. Chauveau is stated as 4,850 to 5,800 cubic *inches*. We cannot regard "cadaveric suggillations" as an improvement on the well-known "post mortem stains," and in one or two other places Dr. Benham has allowed his English to yield somewhat to Gallic influence. In the main, however, the adaptation is fully worthy of the original, and higher praise cannot well be accorded.

The Medical Annual, 1897. (Bristol: John Wright and Co. Price 7s. 6d.)

The Medical Annual has by its success proved how completely it has met a want. It is now in its fifteenth year, and it is no exaggeration to say that every year sees some improvement over the last in its contents or in its arrangement. Constantly engaged as we are in passing under review the medical literature of many countries, we cannot but see in turning over the pages of the Medical Annual how true is the transcript which they give of the improvements and the novelties which have been brought to the notice of the profession during the past year. The work, however, is not a mere medical common place book—a mere series of extracts from the medical literature of the year that has gone by. It is that and something more; for interspersed among its pages are essays by well-known writers which are of real value, giving, as they do, the matured opinion of specialists on the subjects which they have made their own. It commences with a description of the new remedies which have been introduced during the year. After that come upwards of six hundred pages devoted to a detailed description of the various new methods of treatment which have been introduced, alphabetically arranged under the heads of the various diseases dealt with, and interspersed with special essays on such disease as have appeared of particular importance. Then we find a description of the advances made in sanitary science, and of the various new instruments, appliances, and pharmaceutical and dietetic compounds which have recently come into notice. The book is admirably illustrated, and may be confidently recommended as a useful *vade mecum* to those who wish to follow recent advances in medical arts and sciences.

